# Evolution of the ART approach: highlights and achievements

**DOI:** 10.1590/S1678-77572009000700014

**Published:** 2009

**Authors:** Jo E. FRENCKEN

**Affiliations:** DDS, MSc, PhD, Department of Global Oral Health, College of Dental Sciences, Radboud University Nijmegen Medical Centre, The Netherlands.

**Keywords:** Atraumatic Restorative Treatment (ART), Developing countries, Dental caries, Health services research

## Abstract

Atraumatic Restorative Treatment (ART) was initiated in the mid-eighties in Tanzania in response to an inappropriately functioning community oral health programme that was based on western health care models and western technology. The approach has evolved to its present standing as an effective minimal intervention approach mainly because the originators anticipated the great potential of ART to alleviate inequality in oral health care, and because they recognised the need to carry out research to investigate its effectiveness and applicability. Twenty-five years later, ART was accepted by the World Health Organisation (1994) and the FDI World Dental Federation (2002). It is included in textbooks on cariology, restorative dentistry and minimal intervention dentistry. It is being systematically introduced into public oral health service systems in a number of low- and middle income countries. Private practitioners use it. Many publications related to aspects of ART have been published and many more will follow. To achieve quality results with ART one has to attend well-conducted and sufficiently long training courses, preferably in combination with other caries preventive strategies. ART should, therefore, not be considered in isolation and must be part of an evidence-based approach to oral health with a strong foundation based on prevention.

## HISTORY OF EVOLUTION OF THE ART APPROACH

Atraumatic Restorative Treatment (ART) is a minimally invasive approach to both prevent dental carious lesions and stop its further progression. It consists of two components: sealing of carious-prone pits and fissures (ART sealants) and restoration of cavitated dentin lesions with sealant-restorations (ART restorations)^5^. The placement of an ART sealant involves the application of a high-viscosity glassionomer that is pushed into the pits and fissures under finger pressure. An ART restoration involves the removal of soft, completely demineralised carious tooth tissue, using hand instruments. This is followed by restoration of the cavity with an adhesive dental material that simultaneously seals any remaining pits and fissures that remain at risk. In practice the adhesive material predominantly used to restore cleaned cavities produced with hand instruments is a high-viscosity glass-ionomer. Restorations that have used rotary instruments for opening the cavity and hand instruments for cleaning the cavity are not considered ART restorations^7^. These so called modified-ART restorations do not differ from conventional restorations^16^.

ART was initially developed in response to the need to find a method of preserving decayed teeth in people of all ages in underserved communities where resources such as electricity, piped water, conventional dental equipment and finance were rarely available or non-operational. Without this intervention, such teeth would decay further until they were lost through extraction. The approach that ultimately became known as ART was pioneered in the mid-eighties as part of a primary oral health care programme of the Dental School in Dar es Salaam, Tanzania. To support the newly established Dental School, western donors had given ‘mobile’ cast-iron dental chairs, and drill and suction devices. To become operational in rural Tanzania, this equipment required an electrical generator, petrol and a vehicle to transport it. It soon became apparent that the community oral health care training based on the donated “mobile” equipment was impractical and inappropriate. As cited by the students, the lack of finances for running a mobile programme, purchasing spare parts from abroad for the maintenance of the dental equipment and the lack of a vehicle were factors hampering the implementation of a community oral health programme based on the donated equipment.

So, what could be done? Necessity being ‘the mother of invention’, a small investigation was undertaken as to the kind of instruments that were available countrywide in dental clinics in Tanzania. It appeared that hand instruments were available, that most of the dental equipment was non-functional and that zinc-phosphate cement was the only filling material available. Consequently, the management of cavitated dentin lesions was based on the use of hand instruments and available restorative material. In practice such an approach was not found to cause any insurmountable problems, since in many cases the cavity opening was large enough for removal of its soft content; there was no need to use a powerful drill to achieve this. Fracturing thin unsupported enamel in order to open relatively small cavitated dentin lesions with a hatchet was also found possible. In the absence of any proper restorative material, the cleaned cavity was then filled with zincphosphate cement. The patients preferred this manner of treatment to that provided when the donated rotary equipment was used. Following encouraging responses to these early treatments in rural Tanzania, a decision was made to start a pilot study using polycarboxylate cement, rather than zinc-phosphate cement, to fill the cleaned cavities. Evaluation of 28 restorations in children and adults resulted in only one failure after 9 months. In a number of the restorations the polycarboxylate cement was visibly abraded away but the main outcome was that all these people were free of toothache, except for one whose tooth had to be extracted because of pulpitis. However, this cavity was very large before being filled. The enthusiastic patient response and the apparent success of this simple technique were encouraging. The results of the pilot study were presented at the scientific meeting of the Tanzanian Dental Association in 1986, and a minimal intervention approach, later called ART, was officially born.

**Figure 1:**
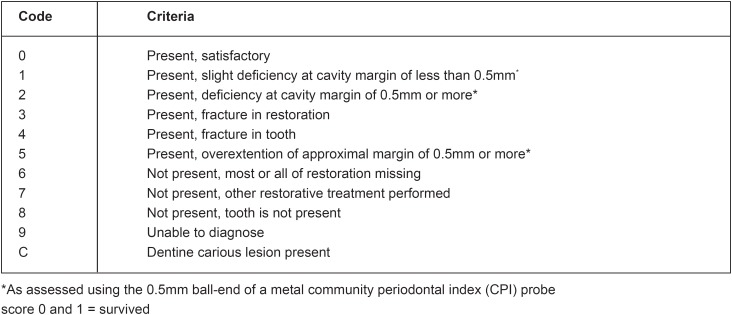
Evaluation criteria for assessing ART restorations

Based on the encouraging results of the pilot study, a field study was started in Tanzania. A permanent restorative material in the form of a medium-viscosity glass-ionomer cement was used instead of polycarboxylate cement. Unpublished results indicated a high level of restoration retention after 3 years. This finding formed the basis for setting up a clinical trial in Thailand in the early nineties, in which the ART approach was compared to the traditional amalgam approach^9^^,^^25^. The first set of ART criteria was developed. These included codes for the expected wear of the medium-viscosity glassionomer used. As material wear was found to be low at the end of the 3-year trial, the first criteria were amended and developed into the currently used ART criteria set (Figure 1).

At the 6^th^-month evaluation of the Thailand study in 1992, it became very apparent that the children who had been treated by ART happily participated, whereas those treated with the traditional rotary hand piece approach were very reluctant to do so. Many of the latter children ran away when they saw the operators, thinking that they needed to be treated again. Both groups of children were asked how they had remembered the treatment from 6 months previously. It became clear that there was a high level of acceptance amongst those treated with ART and an unwillingness to be treated again amongst those in the traditional rotary hand piece group. Hence the term Atraumatic Restorative Treatment (ART) was adopted: “Atraumatic” not only because of its low level of pain or discomfort, but also because of its minimal destruction of tooth tissue.

## HIGHLIGHTS: RESEARCH AGENDA FOR ART

The Thailand study gained attention from world leaders in oral health and resulted in the adoption of ART by the World Health Organization on World Health Day, in 1994. The ART pressrelease from WHO gave high responsibility for ensuring that what was transmitted to the outside world could be proven, to the original ART team consisting of Prof. Taco Pilot, Prof. Prathip Phantumvanit, Dr. Yupin Songpaisan and Dr. Jo Frencken.

Meanwhile, ART studies had started in Cambodia^20^, Zimbabwe^8^ and China^14^. These cohort studies basically investigated the efficacy of ART sealants and ART restorations under field conditions. However, fundamental issues of carious lesion management surfaced as part of the ART studies.

In order to interact with the research community on these fundamental issues, a symposium on Minimum Intervention Techniques for Dental Caries was organised at the 73^rd^ IADR congress in Singapore in 1995. In essence, the meeting was largely devoted to ART and related topics but since the acronym “ART” was not universally known at that time, the title of “Minimal Intervention” was used. It was the 1^st^ ART symposium but under a different name. The most important aspect of the symposium was the development and acceptance of a research agenda on issues related to minimal intervention approaches for caries and, specifically, for ART. A proceeding of the symposium that contained the research agenda was published in the Journal of Dental Public Health in 1996. Setting a research agenda turned out to be of essential importance in stimulating further research related to the ART approach, as a sizable number of researchers based their future research on this agenda.

The 2^nd^ ART symposium took place during the 76^th^ IADR congress in Nice, France in 1998. As in 1995, a proceeding was published; this time in Community Dentistry and Oral Epidemiology, in 1999. It included a paper on the achievements related to the topics of the 1995 research agenda. This paper by Holmgren and Frencken^13^ (1999) assisted many in taking up studies on ART. The 3^rd^ ART symposium took place during 2004-FDI congress in New Delhi but no proceedings were published. The 4^th^ ART symposium was held in Bauru, Brasil in 2004 and the proceedings were published in the Journal of Applied Oral Science in 2006. The 5^th^ ART symposium took place in 2009, during the 3^rd^ Pan Latin America IADR congress in Ilsa de Margarita, Venezuela. All 1^st^ authors of published papers on ART, with workable email addresses, were approached and were asked what they considered to be the future research priorities for ART. The findings have been reported by Holmgren and Figueiredo^12^(2010). By the 1^st^ of December 2009, Pubmed contained 178 published articles on ART, of which 172 are related to the Atraumatic Restorative Treatment approach.

The FDI World Dental Federation set up a committee in 1997 to review the new caries management philosophy of Minimal Intervention Dentistry (MID). The report, describing ART as one of the examples of MID, was published in 2000 in the International Dental Journal and was discussed at the 2002-FDI meeting in Vienna. The General Assembly adopted ART as a minimal intervention approach.

## ACHIEVEMENTS

Many researchers from many countries have investigated different aspects of ART. Some of their findings are listed below:

– Survival rates of single-surface ART restorations using high-viscosity glass-ionomers in primary and permanent posterior teeth are high and meet the American Dental Association (ADA) specifications for quality restorations^29^;
– Survival rates of multiple-surface ART restorations using high-viscosity glass-ionomers in primary posterior teeth do not meet the ADA specifications^29^;
– Survival rates of single-surface ART restorations in permanent posterior teeth, using high-viscosity glass-ionomers, do not differ significantly from comparable traditional restorations using amalgam^10^^,^^23^;
– Survival rates of single-and multiplesurface ART restorations, using high-viscosity glass-ionomers, in primary posterior teeth do not differ significantly from comparable traditional restorations using composite^3^^,^^4^ and compomer^19^;
– Pain felt during treatment was lower in child populations treated restoratively with ART using high-viscosity glass-ionomers, than when traditional restorative procedures were used^15^^,^^21^^,^^25^^,^^26^. Moreover, ART provided without local anaesthesia was better accepted than traditional treatment with local anaesthesia^28^;
– Studies developed to measure dental anxiety contained methodological errors that made it impossible to confirm the hypothesis that ART is less dental anxiety provoking than conventional treatments^17^;
– Initial wear rates of ART restorations using high-viscosity glass-ionomers are low^11^^,^^18^;
– ART restorations using high-viscosity glassionomers were more cost-effective after 2 years than comparable amalgam restorations^23^;
– ART has been introduced in public and private health services of both developing and developed countries and this process is ongoing;
– A chapter on ART has been included in textbooks on Cariology and Minimal Intervention Dentistry;
– ART courses, sometimes in conjunction with other caries-preventive strategies have been conducted in numerous countries.

These outcomes show that the ART approach using high-viscosity glass-ionomers produces quality restorations in single-surface cavities in primary and permanent posterior teeth, which are the cavities most prevalent in most countries. The ART approach saves teeth that otherwise would have to be extracted and prevents carious lesion development. It enhances the opportunity for providing comprehensive basic oral health care for underserved communities, in combination with palliative, preventive and promotional activities (BPOC)^6^. It may also improve the quality of life of patients and the job satisfaction of dentists, particularly those living in underserved communities. In order to achieve all this, dental practitioners have to participate in well-conducted and sufficiently lengthy (at least 5 days) ART courses; preferably in conjunction with other caries preventive strategies.

## CONCLUDING REMARKS

ART is sometimes criticized because it is seen as being merely a restorative treatment performed by dentists. What can restorative care and dentists do to improve oral health in underserved nations? Those asking these questions may have forgotten that early improvement in oral health in Western countries in the 60-70ties occurred because of the presence of preventive and restorative care supported by self-care. They may also not fully understand the philosophy underlying the ART approach. It is not only a restorative but also a preventive and palliative treatment, performed not only by dentists but also by other operating dental personnel, such as dental therapists. This increases the chance for better oral health in underserved communities in both developed and developing countries.

Many dentists see ART as suitable only for developing countries; such as those in Africa where it originated, where many areas lack water and electricity. They do not see it as proper oral care procedure because it does not use sophisticated equipment. ART has its place not only in poor and underserved communities but also in the most exclusive dental practices, as has been reported from countries like the USA^27^, UK^2^ and the Netherlands^1^.

The following may exemplify its potential. I visited a dental clinic in a suburb of Dar es Salaam, Tanzania in August 2009 where ART had been introduced since 2005. One of the dentists told me enthusiastically: “*since I have started work as a dentist in this health centre, now almost 25 years ago, I have never experienced that people come to have their tooth restored. They always come for extraction. But in recent years, they come asking for restorations. I have seen people even come for a check-up, unheard of years ago. This change is due to the education we dentists have received on oral health prevention and, particularly, on the ART approach. I am very happy to still be around to witness the change in oral care after all those many years of pulling teeth”.* She continued: “*the funny thing is that money doesn't seem to matter. They all pay for a restoration which is more expensive than an extraction. What matters for them”,* she said, *“is the fact that teeth now can be restored and that it is done very friendly and pain free”.*

I was profoundly moved by this dentist's statement, remembering the humble beginnings of ART in that country some 25 years ago. Since the birth of ART, the approach has traveled the world. It has boosted the job satisfaction of many dentists and eliminated the suffering of many people. It was also instrumental in showing that by combining effective prevention with a biologically and scientifically based restorative approach it was possible to give hope to improving oral health for the many billions who do not have access to oral care. The fact that the ART team realized the need to engage in proper research from the very start has paid dividends and will continue to do for many years to come.
